# Evaluating markerless biomechanical analysis in a real-world pole vault competition setting

**DOI:** 10.1371/journal.pone.0329987

**Published:** 2026-03-27

**Authors:** Athanassios Bissas, Neil J. Cronin

**Affiliations:** 1 School of Education, Health and Science, University of Gloucestershire, Gloucester, United Kingdom; 2 Neuromuscular Research Centre, Faculty of Sport and Health Sciences, University of Jyväskylä, Jyväskylä, Finland; ASPIRE Academy for Sports Excellence, QATAR

## Abstract

This study evaluated a purpose-trained markerless motion capture system for biomechanical analysis in elite pole vault competition. The aim was to determine whether a markerless approach could produce results comparable to manual digitising—the current standard in live competition settings—when operating under the practical constraint of a fixed four-camera setup. Data were collected from eight world-class pole vaulters during the 2024 World Athletics Indoor Championships. The final steps of the run-up through take-off were recorded at 100 Hz and analysed using both manual digitisation and the SIMI Nemo Markerless system to extract key biomechanical variables. Results showed strong overall agreement between methods for most spatial and centre of mass (CM) variables, with mean relative bias and random error of 0.3% and 3.8%, respectively. Step length differed by approximately 1 cm, and running step velocities showed root mean square error (RMSE) values between 0.02 and 0.05 m/s. CM height and horizontal velocity at pole plant showed RMSEs below 0.02 m and 0.1 m/s, respectively. At take-off, horizontal, vertical and absolute CM velocities all showed RMSE values of approximately 0.1 m/s. For these variables, intraclass correlation coefficients ranged from 0.898–1.000. Continuous waveform agreement was also strong, with Coefficient of Multiple Determination values exceeding 0.98 for vertical CM displacement, and above 0.90 for both CM velocity and most joint angle trajectories. In contrast, joint angles at take-off showed less agreement (RMSE 5°–10°), reflecting challenges in joint landmark identification in field conditions, and indicating that further refinement may be needed for complex movements. These findings suggest that, when supported by anatomically-informed pose estimation algorithms, a four-camera markerless setup is capable of capturing essential performance indicators in elite pole vault. The approach shows strong potential for scientific and applied use in real-world sport environments.

## Introduction

Currently, the most widely used motion analysis technique involves infrared systems that utilise multiple cameras, body markers, and advanced software to capture and analyse movement in three dimensions (3D) in real time. While such technologies have revolutionised research in controlled laboratory environments, their application in live, real-world settings remains problematic. This is primarily due to the uncontrollable nature of competition environments – such as varying weather conditions, lighting, and surface reflections – as well as the impracticality of attaching markers to athletes during live performance, where direct interference by researchers is not feasible.

In contrast, manual analysis of digital video captured via visible light systems continues to be the most common method for studying motion in out-of-lab, competitive environments. Unlike infrared systems, digital videography typically does not require attaching markers to the athlete. However, it relies on the manual identification and tagging (digitising) of key anatomical landmarks in each frame of the video, a process that is highly time-consuming. Manual digitising involves an operator pinpointing these landmarks frame by frame – often at high frame rates and across multiple camera views – to generate 3D motion data. The process is highly labour-intensive, and over prolonged sessions, cognitive fatigue may contribute to occasional inaccuracies. In addition, the time and expertise required make the process financially demanding, potentially limiting its viability for large-scale research projects.

Despite these challenges, manual analyses conducted under strict protocols have consistently delivered precise and trustworthy kinematic data, along with a wide range of performance indicators, to both researchers and coaches over the past several decades [1–6]. Due to its long-standing use, and proven delivery in applied settings, manual digitising continues to be regarded as the dominant method for motion analysis in field-based research. This is particularly true in the sport of athletics, where the vast majority of biomechanical insights have historically been derived from manual digitising studies, including recent analyses involving elite-level athletes [1,6–17]. While this approach has clear strengths, it is not without limitations, particularly when estimating joint angles, where uncertainty around the precise location of internal joint centres can affect accuracy. Importantly, manual analysis also underpins the development of current markerless motion capture techniques, as many supervised machine learning models rely on manually labelled datasets for training and validation. This further reinforces the foundational role of manual digitising in both traditional and emerging approaches to biomechanical analysis.

Recent advances in artificial intelligence have led to the rapid growth of markerless motion analysis techniques, which are rapidly emerging as a key tool in field-based research [18–20]. These approaches use computer vision to interpret visual data from cameras, identifying and tracking human features and body segments across multiple frames. Trained on large datasets, the algorithms can estimate posture, joint angles, and other kinematic variables with increasing precision. Compared to traditional marker-based and manual tagging systems, markerless technologies offer clear advantages: they minimize human-induced variability and can process large volumes of data quickly and efficiently.

Studies investigating markerless motion analysis have shown encouraging accuracy in evaluating human gait and various other movements, although these findings are typically based on relatively simple, single-plane movements such as walking, jumping, or linear sprinting. Recent studies comparing both open-source and commercial markerless systems with multi-camera, marker-based reference systems report average differences for joint angles ranging from 3° to 16°, from 0.1 to 3 m/s for centre of mass (CM) horizontal velocities, and from 2 to 7 cm for linear displacements [21–37]. These values represent total error, incorporating both systematic bias and random variation. While a small number of studies report only the Standard Error of Measurement, which can appear more favourable, this metric reflects only random error and may underestimate actual disagreement. Moreover, in many cases, reported averages were derived across multiple trials, timepoints, or subjects, meaning that higher individual errors may have occurred in specific phases or events but were masked in the aggregated values.

However, it is important to note that all these studies have been conducted under controlled testing conditions, mostly in laboratory settings, where researchers typically have access to numerous cameras and the flexibility to position them optimally, ensuring clear visibility of the relevant body segments throughout the movement.

In more sport-specific, semi-controlled environments, the performance of markerless systems tends to be more variable. For instance, studies in baseball have reported joint angle differences ranging from 4° to 21°, discrepancies in arm speeds of approximately 4 m/s, and average linear displacement errors of 2 cm, with peak errors exceeding 6 cm [38,39]. Similarly, applications of open-source algorithms to football kicking have shown poor agreement with marker-based systems, with mean 3D positional errors ranging from 2 to 9 cm and differences in linear velocities across various landmarks of 1–2 m/s [40].

The discrepancies, both observed in laboratory settings and sport-specific settings may be partly due to the limitations of existing pose estimation algorithms, which are often trained on datasets that do not reflect the complexity or specificity of athletic movements. In many cases, these models rely on pre-defined landmarks that differ from those used in manual or marker-based methods and are annotated by individuals without anatomical or biomechanical expertise [19,20].

These limitations were reflected in our own earlier work [41], which was among the first to apply a markerless pose estimation algorithm in data from a live competitive setting. We tested OpenPose [42,43], a widely used open-source algorithm based on deep learning and predefined anatomical landmarks. When applied to world-class long jump sequences captured during international competition with constrained camera setups, the algorithm produced poor agreement with manual digitising and returned several unrealistic values. These findings reinforced concerns about the suitability of general-purpose models for high-intensity, sport-specific movements under real-world constraints, and highlighted the need for more targeted solutions. More recently, Baker et al. [44] used a custom model to analyse pole vault performance. Although they found some promising results when comparing the model with marker-based motion analysis, they concluded that “joint centre positional inaccuracies observed were problematic, potentially limiting the model’s utility for detailed joint kinematic assessments and competition-performance evaluation”. Thus, there is still scope for improvement in markerless approaches for use in field settings.

Building on our previous findings, the present study represents a further effort to evaluate the capabilities of markerless motion analysis in real-world, competitive athletics environments. Rather than relying on a generic, open-access pose estimation model, this study explores the performance of an event-specific, commercially developed markerless system trained on sports-relevant data. The goal was not to validate any particular software per se, but to assess how well a purpose-trained model could track athletic movement in a setting similar to that of the long jump – namely, the run-up and take-off phases of the pole vault.

This scenario closely mirrors that of our earlier long jump study, both in terms of movement structure and contextual limitations. Although this study benefits from a slightly higher number of cameras (four versus two), the camera configuration remains far from optimal compared to laboratory setups typically involving 8–15 strategically placed units. Camera placement was dictated by on-site logistical constraints during a major athletics championship, limiting the available angles and coverage. As such, the study offers an applied, scenario-specific assessment of markerless motion capture under realistic conditions, where researchers and practitioners often operate with imperfect tools in high-performance environments.

This study addresses a key gap in the relevant literature by applying markerless analysis to high-intensity, elite-level athletic performance – far beyond the basic or constrained movements typically used in laboratory validations – and under the dynamic, uncontrolled conditions of world-class competition. It also represents one of the few applications to date of markerless technology to elite-level sport performance, involving high-speed motion captured under authentic competitive conditions, which are notably different from those used in most existing validation studies.

Therefore, this study aimed to evaluate the performance of a purpose-trained markerless model by comparing its outputs to those of a manually digitised dataset, used here as the reference method. The analysis was based on footage from world-class pole vault performances captured during recent World Championships. Particular focus was placed on performance indicators that are critical to run-up and take-off phases and carry practical significance for both coaches and athletes.

## Materials and methods

### Data collection procedures

Pole vault data were collected from eight finalists (four men and four women) at the 2024 World Athletics Indoor Championships (WIC) in Glasgow. The sample included the men’s three medallists, current and former world champions in both men’s and women’s events, as well as the current men’s world record holder, offering a high-performance cohort for analysis. The analysed attempts were drawn from heights ranging between 5.90 m and 5.95 m for the men, and between 4.55 m and 4.80 m for the women, reflecting the exceptionally high technical standard of the sample. The video footage analysed in this study was recorded during the publicly broadcast pole vault events at the World Athletics Championships in Glasgow by a third-party provider contracted by World Athletics. All participating athletes provided written, informed consent to be filmed and to allow the footage to be used for analysis, as specified in the athlete agreement signed prior to the competition. The footage and associated data are owned by World Athletics, who granted written approval for their use in this study. Although athletes may be visually identifiable in the original footage, no personal or identifying information is included in the manuscript.

**Fig 1:**
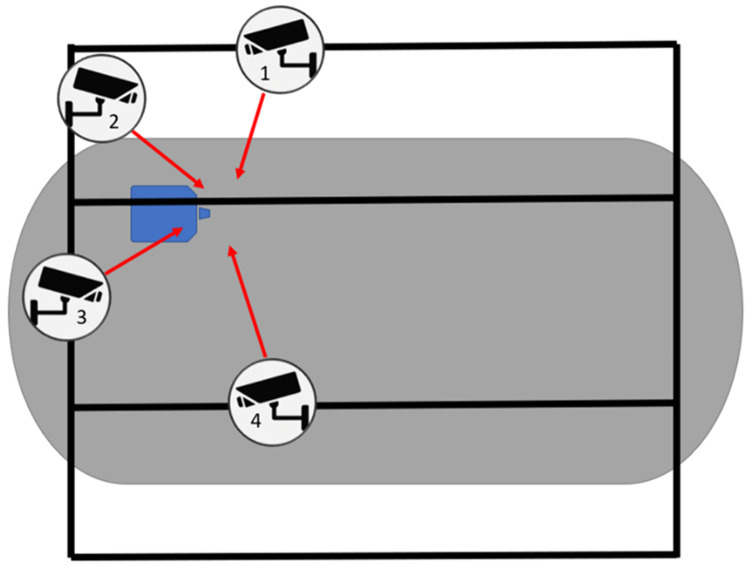
Schematic representation of the 4-camera setup used during data collection. The camera positions are shown relative to the full competition arena (shaded area), with the blue zone indicating the specific location of the pole vault mat and planting box. Camera placement was constrained by the logistical and spatial limitations of the live competition environment.

The jumps were recorded using four Baumer VLXT-31C.I cameras (frame rate: 100 Hz, resolution: 2048 x 1536 pixels) which captured the movement of the athletes during the last phases of the run-up and throughout the pole plant and take-off phases. The cameras were securely mounted on permanent rigging structures fixed to the roof of the arena, and captured movement within the runway section of the pole vault setting (last 12–15 metres from the plant box) from four different angles (Fig 1). The cameras were synchronized via a cable-based Precision Time Protocol setup. This ensured precise frame alignment across all cameras, minimizing temporal discrepancies and allowing for accurate motion tracking and analysis. Calibration was conducted using a wand-based method with active LED markers ensuring precise alignment of the camera system for accurate motion capture. A global coordinate system was established using an active LED L-frame with the calibration residuals (0.000336876 m) exhibiting a high degree of accuracy.

### Manual tracking

Manual digitising was conducted in SIMI Motion (version 10.2.0, Simi Reality Motion Systems GmbH, Germany) to obtain kinematic data during the final three steps and take-off phase of the jump. Ten frames before and after the sequence of interest were also digitised to provide padding for subsequent filtering. In each file and throughout the specified volume, 17 anatomical locations (centre of the head, left and right shoulder, elbow, wrist, metacarpo-phalangeal, hip, knee, ankle and metatarso-phalangeal joint centres) were fully digitised frame-by-frame. Upon completion, adjustments were made using the points-over-frame method to ensure consistency [45]. The manual digitising was carried out by an experienced operator (>500 hours of digitising) who used anatomical criteria alongside the software’s features (e.g., zoom function) to identify the required body landmarks. This methodological approach has previously been shown to display high reliability in competition-based athletics settings [6,14]. The Direct Linear Transformation algorithm was used to reconstruct the 3D coordinates from each camera’s 2D x- and y-coordinates. Reliability of the digitising process was assessed by re-digitising one complete video sequence (over 100 frames) after a 48‑h interval, a time window consistent with standard practice for intra‑operator reliability assessments in biomechanics. As the operator had already logged over 500 hours of digitising experience and followed established protocols used in our group’s previous publications [13–16], this procedure served to confirm repeatability within the specific conditions of the present study rather than to assess general operator competence. The results for critical performance variables demonstrated minimal total error and therefore confirmed the high reliability of the digitising process (Table 1).

**Table 1:** Test- retest reliability of manual digitising.

	RMSE	ICC_3,1_
**Step Length (m)**	0.01	0.999
**CM Vert. Height (m)**	<0.01	0.999
**CM Hor. Velocity (m/s)**	0.02	0.995
**CM Vert. Velocity (m/s)**	0.02	0.992
**Hip Angle Take-off (**^**o**^)	1.4	0.956
**Knee Angle Take-off (**^**o**^)	1.0	0.990
**Ankle Angle Take-off (**^**o**^)	1.9	0.910

RMSE: Root Mean Square Error; ICC: Intraclass Correlation Coefficient.

### Markerless tracking

Markerless tracking was conducted using Simi Nemo Markerless (Simi Reality Motion Systems GmbH, Germany), a motion tracking system that combines non-linear regression modelling and inverse kinematics (IK) for human pose estimation (HPE) without physical markers. The system uses a non-linear regression model that integrates multiple data sources, including Convolutional Neural Network (CNN) extracted key points based on the Simi General Net, 2D silhouette correspondence, and joint constraints, to generate an initial pose estimate. Joint positions are then refined through feature fusion and pose optimisation. IK is subsequently applied to compute the most plausible pose configuration for each segment. The system employs an extended IK model based on the Humanoid Animation (H-Anim) standard, incorporating 25 articulated segments to represent the human body.

### Data extraction

SIMI Motion (10.2.0) and MATLAB (version R2023a, MathWorks, Inc., Natick, MA) were used for data processing, filtering and extraction for both approaches. A 20 Hz low-pass, recursive, second-order Butterworth filter was applied to both datasets to ensure consistent processing; this value matched the default used by the markerless system and closely aligned with the expected optimal range for the manual data. Matching anatomical features and joint reference landmarks to the manual analysis were identified and extracted from SIMI Nemo. In addition to individual tracked points, a de Leva based segmental model [46] was created, allowing segment and whole-body CM variables to be calculated. Regarding performance data, a number of relevant biomechanical variables were calculated during the run-up and at two critical events (Tables 2 and 3). For both systems, the key events were manually identified based on visual inspection of the synchronised video frames, using consistent definitions to ensure temporal alignment between datasets.

**Table 2:** Distinct events selected to analyse the performance of the athletes.

EVENT	DEFINITION
**Pole Plant**	The time instant when the pole contacts the back of the plant box.
**Take-off**	The last point of contact when the foot leaves the runway.

**Table 3:** Biomechanical variables selected to describe the performance of the athletes.

VARIABLE	DEFINITION
**Average (Avg) Runway Velocity**	The mean Centre of Mass (CM) horizontal velocity achieved during the last three steps of the run-up.
**Maximum (Max) Runway Velocity**	The maximum CM horizontal velocity achieved during the last three steps of the run-up.
**3**^**rd**^ **Last Step Length**	The toe-off to toe-off distance of the third last step before take-off.
**3**^**rd**^ **Last Step Velocity**	The mean CM horizontal velocity during the third last step before take-off.
**2**^**nd**^ **Last Step Length**	The toe-off to toe-off distance of the step immediately before the last step.
**2**^**nd**^ **Last Step Velocity**	The mean CM horizontal velocity during the step immediately before the last step.
**Last Step Length**	The toe-off to toe-off distance of the step immediately before take-off.
**Last Step Velocity**	The mean CM horizontal velocity during the step immediately before take-off.
**CM Height at Pole Plant (PP)**	The vertical distance between the runway and the CM at pole plant.
**CM Hor. Velocity at PP**	The instantaneous CM horizontal velocity at the moment of pole plant.
**CM Vert. Velocity at PP**	The instantaneous CM vertical velocity at the moment of pole plant.
**Standing Height**	The vertical distance between the runway and the CM at take-off.
**Hip Angle**	The angle between the trunk and thigh segments and considered to be 180° in the anatomical standing position.
**Knee Angle**	The angle between the thigh and lower leg segments and considered to be 180° in the anatomical standing position.
**Ankle Angle**	The angle between the lower leg and foot segments.
**CM Hor. Velocity at Take-off**	The instantaneous CM horizontal velocity at the moment of take-off.
**CM Vert. Velocity at Take-off**	The instantaneous CM vertical velocity at the moment of take-off.
**CM Abs. Velocity at Take-off**	The instantaneous CM resultant velocity at the moment of take-off.
**CM Take-off Angle**	The angle between the path of the CM and the horizontal at take-off.

The take-off leg (TOL) was defined as the last leg that contacts prior to the body leaving the ground, whilst the drive leg (DL) was defined as the leg that swings forward and upward as the athlete leaves the ground; Joint angles were calculated using the joint centres and the longitudinal axes of adjacent body segments, and projected onto the sagittal plane of the global coordinate system.

### Statistical analysis approach

Manual analysis was considered as the reference (REF) method and the markerless (ML) as the alternative/new method, being evaluated against the accepted standard REF. Both methods were tested on the same mechanical and performance variables by placing the emphasis on agreement statistics. All statistical analyses were conducted in MATLAB and SPSS (version 29, IBM, NY). Comparisons between the two techniques for discrete (zero-dimensional) data, such as average and instantaneous velocities, lengths, and postural characteristics, were performed as follows: Limits of Agreement (LOA) incorporating Bias and Random Error were constructed to assess agreement between the two methods; Root Mean Square Error (RMSE) was used to provide a level of difference between the ML and the REF method; Intraclass correlation coefficients (ICC 3,1) were calculated to provide a measure of relative reproducibility between the two techniques.

Agreement between methods for time-series CM and joint angle data between the touchdown (TD) of the third last step and the take-off (TO) instant, was assessed with a coefficient of multiple determination (CMD) using the in-built MATLAB function ‘fitlm’, as used previously to display reliability of kinematic data [47]. CMD quantifies the waveform similarity between two curves, with values ranging between zero (highly dissimilar waveforms) and one (highly similar waveforms). Therefore, although it accounts for similarity in curve shape, it does not consider the amplitude of the waveform. CMD and ICC (3,1) values were interpreted as: 0.00–0.50 = “poor”; 0.50–0.75 = “moderate”; 0.75–0.90 = “good”; and 0.90–1.00 = “excellent” based on the guidelines of Koo and Li [48] for ICC interpretation.

## Results

Central tendency and variability for discrete biomechanical variables are expressed as mean ± standard deviation (SD), providing a quantitative overview of group-level performance (Table 4). Values are reported for the full sample (n = 8) as well as disaggregated by sex, with separate statistics for male (n = 4) and female (n = 4) athletes. This breakdown allows for initial comparisons between subgroups while also capturing the range of technical execution observed within the cohort.

**Table 4:** Mean ± SD for biomechanical variables from Glasgow WIC 2024, using both analysis methods.

	ALL		MEN		WOMEN	
	REF	ML	REF	ML	REF	ML
**Avg Runway Velocity (m/s)**	8.96 ± 0.77	8.96 ± 0.78	9.63 ± 0.28	9.64 ± 0.28	8.30 ± 0.35	8.29 ± 0.37
**Max Runway Velocity (m/s)**	9.21 ± 0.79	9.29 ± 0.78	9.90 ± 0.29	9.97 ± 0.30	8.53 ± 0.32	8.61 ± 0.34
**3**^**rd**^ **Last Step Length (m)**	2.09 ± 0.13	2.09 ± 0.14	2.20 ± 0.08	2.20 ± 0.10	1.98 ± 0.04	1.98 ± 0.05
**3**^**rd**^ **Last Step Velocity (m/s)**	8.92 ± 0.78	8.92 ± 0.81	9.60 ± 0.30	9.61 ± 0.30	8.25 ± 0.36	8.22 ± 0.41
**2**^**nd**^ **Last Step Length (m)**	2.06 ± 0.19	2.06 ± 0.18	2.20 + 0.12	2.20 ± 0.11	1.92 ± 0.13	1.92 ± 0.12
**2**^**nd**^ **Last Step Velocity (m/s)**	8.96 ± 0.79	8.96 ± 0.78	9.63 ± 0.31	9.63 ± 0.30	8.29 ± 0.38	8.29 ± 0.38
**Last Step Length (m)**	1.89 ± 0.17	1.89 ± 0.18	1.96 ± 0.15	1.97 ± 0.16	1.81 ± 0.18	1.82 ± 0.18
**Last Step Velocity (m/s)**	9.01 ± 0.74	9.03 ± 0.75	9.66 ± 0.26	9.68 ± 0.24	8.36 ± 0.31	8.37 ± 0.33
**CM Height at PP (m)**	1.09 ± 0.05	1.07 ± 0.06	1.12 ± 0.02	1.11 ± 0.02	1.06 ± 0.06	1.04 ± 0.06
**CM Hor. Velocity at PP (m/s)**	8.38 ± 0.75	8.35 ± 0.80	8.92 ± 0.48	8.93 ± 0.52	7.83 ± 0.53	7.78 ± 0.59
**CM Vert. Velocity at PP (m/s)**	0.84 ± 0.64	0.90 ± 0.58	1.11 ± 0.68	1.11 ± 0.64	0.57 ± 0.55	0.69 ± 0.50
**Standing Height (m)**	1.21 ± 0.06	1.20 ± 0.06	1.25 ± 0.02	1.24 ± 0.02	1.18 ± 0.06	1.17 ± 0.06
**DL Hip Angle at Take-off (**^**o**^)	141.43 ± 16.81	145.61 ± 17.92	140.06 ± 13.13	141.39 ± 14.92	142.80 ± 21.96	149.84 ± 21.90
**DL Knee Angle at Take-off (**^**o**^)	55.38 ± 10.58	58.39 ± 11.37	59.82 ± 9.90	58.96 ± 13.17	50.95 ± 10.54	57.83 ± 11.29
**DL Ankle Angle at Take-off (**^**o**^)	122.45 ± 9.08	119.16 ± 10.19	120.39 ± 8.98	121.26 ± 6.65	124.50 ± 10.03	117.06 ± 13.65
**TOL Hip Angle at Take-off (**^**o**^)	194.15 ± 4.35	202.82 ± 5.96	193.35 ± 5.84	204.59 ± 5.98	194.35 ± 3.13	201.04 ± 6.23
**TOL Knee Angle at Take-off (**^**o**^)	167.25 ± 4.09	170.40 ± 3.45	168.02 ± 5.76	170.52 ± 3.33	166.49 ± 2.10	170.28 ± 4.08
**TOL Ankle Angle at Take-off (**^**o**^)	131.74 ± 6.06	122.73 ± 4.08	133.11 ± 4.51	122.95 ± 4.28	130.37 ± 7.77	122.51 ± 4.51
**CM Hor. Velocity at Take-off (m/s)**	7.45 ± 0.67	7.37 ± 0.66	8.02 ± 0.24	7.90 ± 0.29	6.88 ± 0.36	6.85 ± 0.42
**CM Vert. Velocity at Take-off (m/s)**	2.48 ± 0.29	2.50 ± 0.21	2.70 ± 0.19	2.64 ± 0.22	2.26 ± 0.16	2.37 ± 0.10
**CM Abs Velocity at Take-off (m/s)**	7.85 ± 0.70	7.79 ± 0.66	8.46 ± 0.23	8.34 ± 0.26	7.25 ± 0.37	7.25 ± 0.39
**CM Take-off Angle (**^**o**^)	18.38 ± 1.25	18.80 ± 1.52	18.61 ± 1.47	18.47 ± 1.74	18.16 ± 1.17	19.13 ± 1.44

Note: CM: Centre of Mass; PP: Pole Plant; TOL: Take-off Leg; DL: Drive Leg.

**Fig 2:**
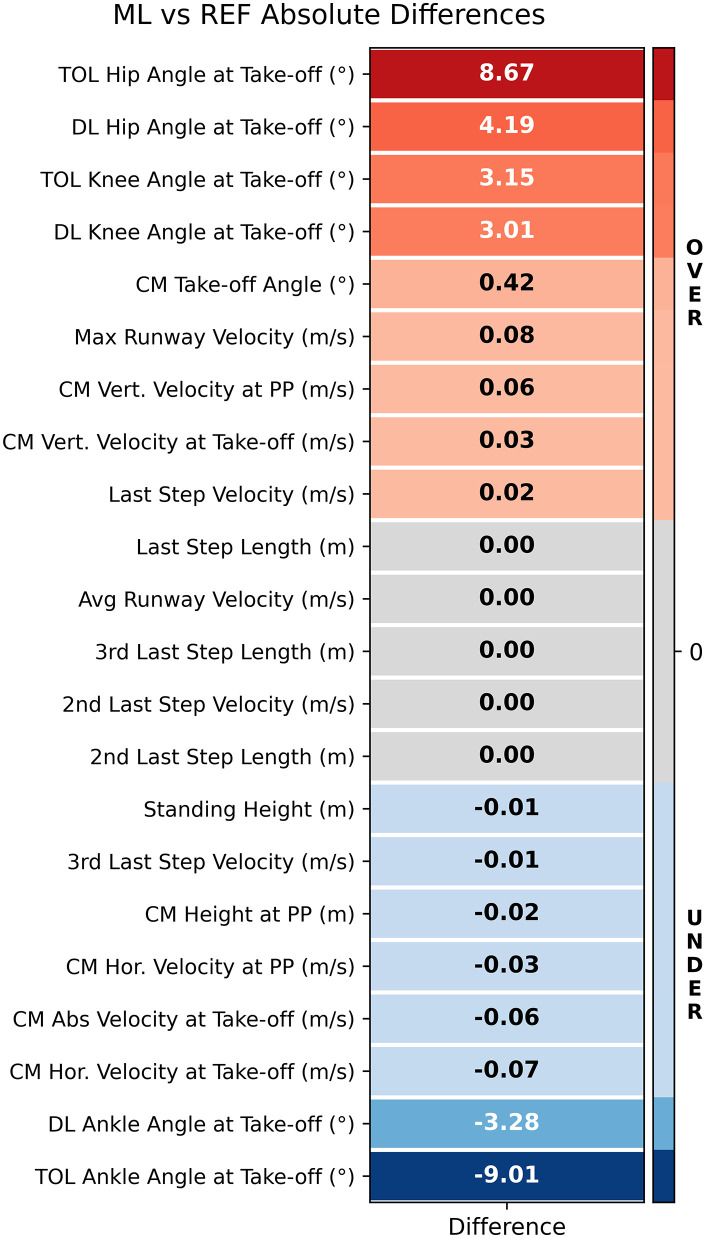
Heatmap showing absolute differences between the markerless (ML) method and the marker-based reference (REF) method for each measured variable. The colour scale represents both the magnitude and direction of the difference, with warmer tones indicating instances where ML reports higher (overestimating) values than REF, and cooler tones indicating the opposite (underestimating). Values represent the mean difference for each variable across participants.

The observed absolute differences between the REF and ML methods across all biomechanical variables are visually represented in a heatmap (Fig 2), allowing for a concise comparison of the magnitude and direction of these differences across measurement types, with warmer and cooler tones indicating positive and negative deviations, respectively. This comparison shows that the largest discrepancies were observed in distal joint angles, particularly at the ankle (up to 9°), while variables related to whole-body CM kinematics, such as vertical velocity or position, showed comparatively smaller differences. The directionality of the differences also varied, with some variables tending to be underestimated and others overestimated by the ML method relative to the REF.

**Fig 3:**
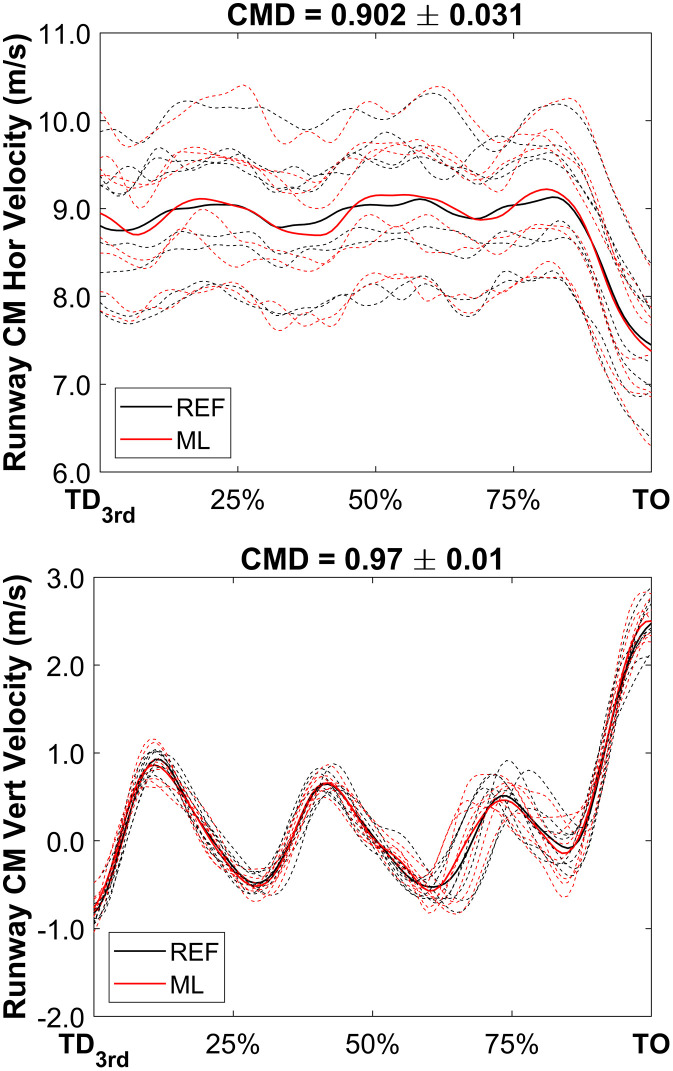
CM runaway velocity waveforms (from 3rd last step touchdown to the take-off instant) for the whole group (n = 8). Black lines represent the REF method and red lines represent the ML method, whilst solid lines represent group mean data and dashed lines athlete individual data. CMD values above each subplot show waveform similarity.

**Fig 4:**
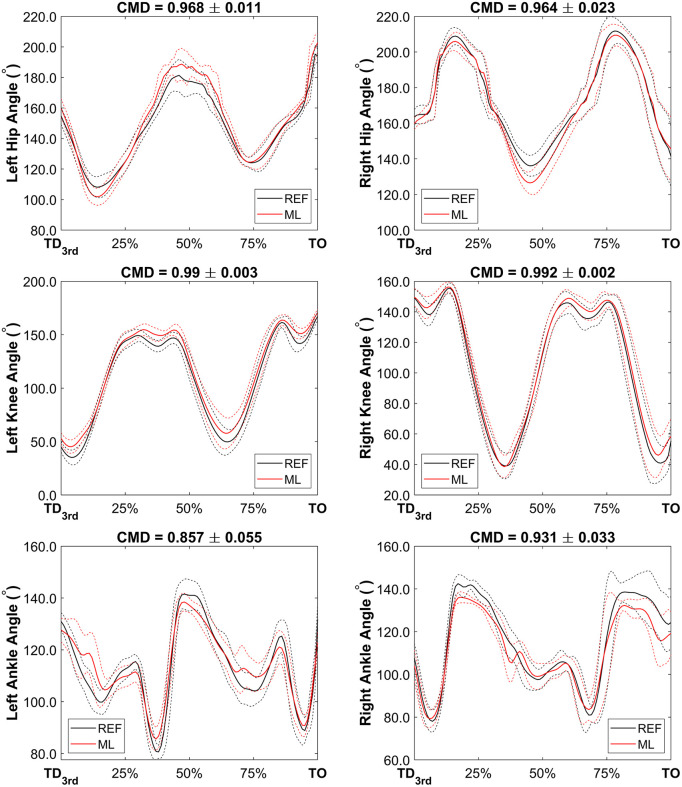
Joint angle waveforms (from 3rd last step touchdown to the take-off instant) for the whole group (n = 8). Black lines represent the REF method and red lines represent the ML method, whilst solid lines represent group mean data and dashed lines group SD data. CMD values above each subplot show waveform similarity.

CMD values across the continuous data variables showed a high degree of similarity. A perfect agreement (1 ± 0) was observed for the horizontal displacement of the CM, while the vertical and mediolateral displacement exhibited values of 0.982 ± 0.008 and 0.935 ± 0.079 respectively. CM runaway velocity waveforms showed excellent agreement, 0.902 ± 0.031 for horizontal and 0.970 ± 0.010 for vertical direction. Finally, waveform agreement across joint angles for both legs varied from good (0.857 ± 0.055 for Left Ankle) to excellent (0.992 ± 0.002 for Right Knee). Figs 3 and 4 present time-series waveform comparisons between the REF and ML methods for selected variables. For each plot, both individual trials and group-level summaries are displayed, with CMD values included as indicators of waveform similarity. All athletes performed take-off using their left leg.

Results concerning agreement between methods for discrete parameters are presented in Table 5. Bias, random error and RMSE values for spatial and most CM related measures were very low with ICC values approaching 1.0. In contrast, joint angle variables showed comparatively poorer agreement, with LOA driven by both systematic and random error components.

**Table 5:** Agreement statistics for discrete biomechanical variables between REF and ML methods (n = 8).

	Bias	Random Error	LOA	RMSE	ICC (3,1)
**Avg Runway Velocity (m/s)**	0.00	0.04	−0.04–0.04	0.02	1.000
**Max Runway Velocity (m/s)**	−0.08	0.10	−0.18–0.02	0.09	0.993
**3**^**rd**^ **Last Step Length (m)**	0.00	0.02	−0.03–0.02	0.01	0.997
**3**^**rd**^ **Last Step Velocity (m/s)**	0.01	0.10	−0.09–0.11	0.05	0.998
**2**^**nd**^ **Last Step Length (m)**	0.00	0.01	−0.01–0.01	0.01	0.999
**2**^**nd**^ **Last Step Velocity (m/s)**	0.00	0.06	−0.06–0.06	0.03	0.999
**Last Step Length (m)**	0.00	0.01	−0.01–0.01	0.01	0.999
**Last Step Velocity (m/s)**	−0.02	0.05	−0.07–0.04	0.03	0.999
**CM Height at PP (m)**	0.02	0.01	0.00–0.03	0.02	0.943
**CM Hor. Velocity at PP (m/s)**	0.02	0.21	−0.19–0.23	0.10	0.991
**CM Vert. Velocity at PP (m/s)**	−0.06	0.29	−0.35–0.23	0.15	0.969
**Standing Height (m)**	0.01	0.01	0.00–0.02	0.01	0.980
**DL Hip Angle at Take-off (**^**o**^)	−4.19	11.85	−16.03–7.66	7.04	0.920
**DL Knee Angle at Take-off (**^**o**^)	−3.01	14.24	−17.25–11.23	7.43	0.773
**DL Ankle Angle at Take-off (**^**o**^)	3.28	12.35	−9.07–15.64	6.75	0.763
**TOL Hip Angle at Take-off (**^**o**^)	−8.67	6.44	−15.11– − 2.23	9.20	0.340
**TOL Knee Angle at Take-off (**^**o**^)	−3.15	7.21	−10.35–4.06	4.66	0.410
**TOL Ankle Angle at Take-off (**^**o**^)	9.01	7.91	1.10–16.92	9.77	0.280
**CM Hor. Velocity at Take-off (m/s)**	0.07	0.16	−0.08–0.23	0.10	0.988
**CM Vert. Velocity at Take-off (m/s)**	−0.03	0.23	−0.26–0.21	0.11	0.898
**CM Abs Velocity at Take-off (m/s)**	0.06	0.19	−0.13–0.25	0.11	0.980
**CM Take-off Angle (**^**o**^)	−0.42	1.68	−2.09–1.26	0.90	0.790

Note: LOA: Limits of Agreement; RMSE: Root Mean Square Error; ICC: Intraclass Correlation Coefficient; PP: Pole Plant; TOL: Take-off Leg; DL: Drive Leg; CM: Centre of Mass *LOA were calculated using data to 3dp.

## Discussion

The results indicate a high level of agreement between REF and ML across most variables, with noticeable deviations observed in discrete joint angles. Considering the test–retest reliability of the manual method (Table 1), the level of agreement observed between the markerless and manual approaches for spatial and CM variables suggests that, for these measures, the markerless system performs within the expected range of measurement variability. This supports its potential use as an interchangeable tool for out-of-the-lab applications.

For all runway variables, systematic differences (bias) were approximately zero, indicating an excellent fundamental level of agreement between methods. Random errors for these variables were also very low, resulting in narrow limits of agreement. RMSE values showed a 1 cm difference for step length and ranged from 0.02 to 0.05 m/s for average and step velocities, reflecting a close correspondence between methods. Maximum running velocity displayed a slightly higher difference, as expected for an instantaneous measurement, but the observed deviation of 0.09 m/s – approximately 1% of an average velocity of 9.20 m/s – is considered of trivial practical significance. These consistent levels of agreement are further supported by ICC coefficients approaching 1.0.

Regarding critical instantaneous variables, agreement between the two methods was particularly close for CM height and horizontal velocity at pole plant, with slightly greater variation observed for CM vertical velocity at the same instant. ICC values exceeding 0.9 confirm this level of consistency and support the potential of the markerless approach in future applications.

Key variables measured at take-off showed strong agreement between methods for horizontal and absolute CM velocities, with RMSE values around 0.1 m/s and limits of agreement not exceeding 0.25 m/s. CM vertical velocity also demonstrated close agreement, although it was associated with slightly higher random error. Standing height differences were minimal, with deviations typically within 1 cm. The CM take-off angle exhibited an average RMSE below 1°, with maximum unidirectional differences of no more than 2°. All values were accompanied by high ICCs, indicating that the two methods produced comparable results and may be considered interchangeable for these parameters.

In contrast, joint angle measurements at take-off demonstrated only limited to moderate agreement. RMSE values ranged from 5° to 10° across the various angles, with only the drive leg producing ICC values above 0.75. While both systems relied on the same anatomical landmarks, the lower agreement in joint angles may stem from underlying differences in how those landmarks are interpreted, in particular whether the interpretation of those landmarks by the machine learning model aligns precisely with expert manual digitisation. Manual digitisation allows the operator to draw on anatomical knowledge, temporal context, and frame-by-frame refinement, whereas the markerless algorithm operates in near real time without human oversight. Further investigation is needed to better understand the sources of variability in joint angle estimation under field conditions. More broadly, these results highlight a fundamental challenge in estimating joint angles during real-world markerless competition analysis. Whether using manual or automated techniques, the precise identification of external joint landmarks – particularly when athletes are wearing competition clothing, and when views are partially obstructed – remains inherently difficult. It is important to note that anatomical landmarks used in motion analysis are only proxies for underlying joint centres, which cannot be directly observed. While laboratory marker-based systems typically offer the most precise estimations of joint centre positions, even these are subject to limitations. In the current study, neither the manual nor the markerless method employed physical markers, making accurate joint centre identification particularly challenging. It is possible that employing a greater number of cameras would have improved landmark visibility and provided the markerless algorithm with more comprehensive visual data to support joint estimation. In addition, the asymmetrical nature of upper body movements in pole vault, particularly between the dominant and non-dominant arms during the run-up and take-off, may contribute to further variability in estimating lower body angles such as those at the hip, since shoulder position influences these calculations. This challenge is amplified in outdoor, dynamic, multi-planar environments, where ideal conditions for visibility and landmark estimation cannot be guaranteed. While ongoing methodological improvements are essential and will undoubtedly enhance accuracy, some level of uncertainty may always remain when estimating joint angles without physical markers in such settings.

Finally, the CMD values for the CM variables showed an excellent degree of similarity (>0.9), supporting the findings for the discrete CM variables. Interestingly, all CMD values for the angles ranged from good to excellent, a promising observation for future refinement of the tracking method. The high CMD values across the variables indicate strong overall similarity in the temporal patterns of the compared curves. The CMD metric reflects the proportion of variance in one waveform that is explained by another across the entire time series. As such, CMD is sensitive to global trends but less responsive to localized deviations. Consequently, high CMD values can coexist with substantial differences at specific discrete points, especially if these deviations are temporally narrow or occur in regions of low signal variance. This highlights the importance of complementing CMD with pointwise analyses when evaluating waveform agreement.

This study contributes to an underrepresented area within the current body of research, as most prior comparisons between marker-based and markerless motion capture systems have been conducted in controlled laboratory environments, typically evaluating open-source or commercial markerless approaches against optoelectronic systems. To the authors’ knowledge, only one published study has compared markerless analysis with manual digitising in a comparable real-world context, using a limited number of cameras in line with the practical constraints of a competition environment. In that investigation – also conducted by the present research team – long jump sequences were analysed using both manual digitisation and the OpenPose markerless system [41]. The findings indicated that OpenPose did not achieve the same level of accuracy as manual methods for capturing reliable kinematic data in a competitive setting. More recently, Baker et al. [44] also examined markerless tracking in pole vault, but their work was conducted in an indoor training context with more controlled capture conditions. While relevant, their findings highlighted significant joint centre inaccuracies, and the environment was not fully representative of live competition settings. As such, the present study provides a complementary and novel perspective by evaluating a purpose-trained markerless model during actual world-class competition, under the real-world constraints of a major indoor championship.

Previous validation studies have demonstrated that markerless motion capture systems can achieve reasonable levels of accuracy under controlled laboratory conditions, particularly when analysing relatively simple and constrained movement tasks [21–37]. In these environments, researchers typically benefit from unobstructed camera views, consistent lighting, and optimal positioning of a large number of cameras – factors that collectively enhance pose estimation quality. These conditions tend to produce relatively modest differences between markerless and marker-based outputs, particularly for gross kinematic measures. While such levels of error may be acceptable for general descriptions of movement, they may still be too large when more nuanced performance characteristics are under investigation. However, when these systems are applied in more complex, sport-specific settings, their performance tends to become more variable [38–40]. Movements in such contexts are often multidirectional, faster-paced, and less constrained, and camera placement is typically limited by logistical or spatial constraints. As a result, the reliability and precision of markerless tracking can be compromised, particularly for subtle biomechanical variables.

The present study differs from many previous investigations in that it compares a markerless motion capture system to manual digitising rather than to a marker-based, optoelectronic reference. While manual digitising is generally associated with lower spatial accuracy and somewhat reduced repeatability compared to marker-based systems, it remains one of the few viable options for conducting full biomechanical analyses in live competition settings, where marker placement and complex equipment setups are not feasible. It also plays a foundational role in training markerless algorithms, many of which rely on manually annotated datasets for supervised learning. Another distinguishing feature of this study is the use of a purpose-trained markerless model, developed using real-world, sports-relevant data. This tailored approach enhances the model’s relevance to the applied setting and represents an important advancement over more generic or pre-trained systems. In addition, data collection was carried out in an outdoor competition environment involving dynamic, unconstrained movements, which introduces greater variability than typically seen in laboratory-based studies. These conditions offer a practical context for evaluating markerless motion capture techniques and contribute to a broader understanding of their applicability in performance-focused field environments.

From a real-world performance perspective, the data obtained in the current study compare favourably with previously published findings from similar competitive environments. These earlier analyses, conducted using the same reference method and software, include findings from the 2017 World Championships in London and the 2018 World Indoor Championships in Birmingham [49–53]. These investigations have contributed one of the most extensive biomechanical datasets on pole vault performance to date, employing high-speed 3D motion capture techniques in live international competition settings. Notably, several athletes included in the present dataset also participated in the 2017 and 2018 studies, offering a valuable opportunity for informal longitudinal comparison.

It is important to emphasise that comparisons with previous studies are not intended to establish direct equivalence, but rather to assess whether the biomechanical values observed here fall within the expected range for elite pole vault performance. At the highest level, athletes tend to exhibit broadly similar movement patterns due to the shared mechanical demands of the event, though individual variation remains essential. Technique is adapted based on factors such as physical characteristics, jump height, and competition context, introducing natural variability both between and within athletes.

In cross-study comparisons, differences in data collection conditions such as camera placement, number of views, or environmental factors can also contribute to variation in measured values. While these factors do not undermine the overall biomechanical patterns, they should be considered when interpreting results across settings. Overall, although this study was not designed to replicate previous analyses, the consistency of the current markerless data with prior benchmarks supports its credibility. Importantly, the RMSE values observed in the present study were generally comparable to, and often smaller than performance-related differences reported in the pole vault literature [49,50,53–55]. For example, average velocity changes of 0.10 to 0.90 m/s have been observed between successive segments of the approach run in large samples of vaulters across performance levels. Between-group and within-athlete variations in approach velocity among elite finalists have been reported in the range of 0.09 to 0.39 m/s, while step length differences between groups (e.g., junior vs. senior athletes) often fall between 0.07 and 0.12 m. Intra-athlete variability (e.g., between successful and unsuccessful attempts) has also shown step length differences averaging around 7 cm. In contrast, our RMSE values were consistently below 0.10 m/s for runway and step velocities and around 1 cm for step lengths. Similarly, CM positional errors remained within 1–2 cm. These findings indicate that the markerless method used here was sufficiently sensitive to detect meaningful performance differences, both between and within athletes, thereby reinforcing its potential for use in high-performance analysis settings, including live competition environments.

While the findings add to a growing body of evidence supporting the use of markerless motion capture in applied sport contexts, several methodological considerations should be acknowledged to contextualise the results. The sample size was limited by access to elite-level participants during a live competition; however, the within-subject comparison design and inclusion of complementary agreement metrics (ICC, RMSE, LOA) provide confidence in the reliability of the results. Although camera placement met the requirements for 3D reconstruction, some viewing angles were less than optimal for consistently identifying anatomical landmarks during complex phases of the vault. Structural elements of the competition setup, such as the uprights supporting the crossbar, also partially obstructed the field of view at key moments, occasionally affecting landmark detection. The large capture volume necessary for the event led to a trade-off in effective resolution for finer details, despite the use of high-quality cameras. Moreover, the number of cameras was limited to four, dictated by logistical and practical constraints inherent to live competition environments. While this configuration was sufficient for capturing key variables, a greater number of cameras would theoretically improve spatial coverage and landmark visibility for both manual and markerless analysis. These factors, inherent to in-situ biomechanics, applied to both the markerless and reference methods and are unlikely to have introduced systematic bias. Some variables related to pole plant and take-off could not be analysed, as the pole tip was not consistently visible. Additionally, variability in athlete clothing and background contrast may have influenced the reliability of landmark detection. Together, these considerations reflect the practical challenges of live biomechanical analysis and underscore the need for ongoing refinement as markerless techniques are further developed for elite sport applications. In summary, whilst it was not feasible to isolate the individual contribution of each potential error source, we followed established principles of motion capture and calibration to minimise their impact, and we are confident that data collection was carried out to a standard consistent with long-standing practices in applied sport biomechanics.

A final consideration relates to the proprietary nature of the markerless system used in this study. The internal HPE, multi-view reconstruction, and IK components are vendor-defined and not publicly disclosed. While this limits full algorithmic transparency, the aim of the present study was to empirically evaluate system-level agreement against manual digitisation under real-world competition conditions, rather than to validate the internal modelling architecture. Such validation-based assessment of commercially available systems through performance metrics (e.g., RMSE, ICC, LOA) is consistent with common practice in applied biomechanics. Within contractual and intellectual property constraints, we have described the acquisition setup, processing parameters, and comparison framework in sufficient detail to support methodological replication using the same configuration.

## Conclusions

This study represents a meaningful step in evaluating the use of a markerless motion capture system within the demanding context of elite pole vault competition. By comparing outputs against an established manual reference, the system demonstrated generally strong agreement in most biomechanical variables, even under the constraints of outdoor, real-world conditions. Key variables such as running velocity, step lengths, CM kinematics, and take-off characteristics showed very good to excellent agreement between methods, with differences remaining small from both a statistical and practical perspective. Nevertheless, certain limitations were evident, particularly in the estimation of joint angles at take-off, where factors such as occlusion, clothing variability, and uncertainty in joint landmark identification may influence automated tracking more than manual annotation. Based on these findings, a four-camera configuration of the kind used in this study – when supported by purpose-trained markerless tracking algorithms and pose estimation software – appears sufficient for accurately capturing critical performance variables in pole vault, making it a viable tool for both scientific investigation and applied support for coaches and athletes. The ability to capture high-quality kinematic data without markers in live competition settings represents a promising direction for future research and performance analysis in sport.
